# Access to Gender-Affirming Care and Alternatives to That Care Among Transgender Adults

**DOI:** 10.1001/jamanetworkopen.2025.20808

**Published:** 2025-07-16

**Authors:** Teresa A. Graziano

**Affiliations:** 1 College of Nursing and Health Science, Department of Nursing, University of Vermont, Burlington

## Abstract

This survey study examines alternatives that transgender adults may consider in place of monitored gender-affirming care, if such care becomes unavailable.

## Introduction

Health equity for 1.6 million transgender, 1.2 million nonbinary, and approximately 5 million intersex US individuals (hereafter, *gender minority individuals*) has improved as clinicians recognize their unique health challenges.^1^ Gender minority individuals may access gender-affirming care (GAC) for evidence-based services like hormone therapy, surgical procedures, and mental health care. Research has demonstrated that prompt access to GAC reduces anxiety, depression, and suicidal ideation.^1,2,3,4^ For example, of 92 329 gender minority US individuals who received gender-affirming hormones and gender-affirming surgery, 98% and 97%, respectively, report increased life satisfaction.^1^

Despite strong evidence that GAC is beneficial and improves patient lives,^1,2,3,4^ President Donald J. Trump signed an executive order directing agencies to restrict GAC access for individuals younger than 19 years.^5^ Clinicians play a crucial role in safe hormone prescribing, monitoring adverse effects, and preventing reliance on risky alternatives like do-it-yourself hormone therapy. Given the uncertain future of GAC, understanding alternatives that gender minority individuals are considering in place of monitored GAC is critical.

## Methods

This survey study was approved by the University of Vermont institutional review board, which granted an informed consent waiver, in accordance with 45 CFR §46.104(d)(2). Participants permitted sharing deidentified responses. This study follows STROBE guidelines. Expanded methods are shown in the eAppendix in Supplement 1. Gender minority participants who were aged 18 years or older, English-speaking, and resided in the US were recruited from social media (ie, Tumblr, Reddit, Facebook, Bluesky, and Instagram) to complete an anonymous survey from December 2, 2024, to January 20, 2025. Participants self-reported their demographics and were presented with two open-response questions. Item 1 asked, “Do you think you will lose access to gender affirming care in the next four years?” Item 2 asked, “If you find yourself unable to receive gender affirming care, what alternative options for care are you aware of, or planning to take?”

Preliminary data were stored in an SPSS version 29.0.2 (IBM) file. For item 1, submissions were read in total and coded as either believing GAC will be lost or not. Participants who reported do-it-yourself hormones or death as alternatives to GAC were counted. Of the responses that discussed death, some shared active suicidal ideation, while others were passive. For this reason, responses that considered death as an alternative were counted separately as either active or passive suicidal ideation.

## Results

In total, 489 gender-minority adults (mean [SD] age, 25.6 [6.3] years; age range, 18-64 years; 337 assigned female at birth [68.9%]) of the 690 participants who began the survey completed open-response items 1 and 2. See the Table for sociodemographics and the Box for representative quotations. All 489 participants reported they believed their access to GAC would be restricted in the next 4 years. In item 2, 155 participants (31.7%) reported they would consider using do-it-yourself hormone therapy as an alternative if GAC is further restricted. Thirty-two participants (6.5%) expressed active suicidal ideation, and 72 participants (14.7%) expressed passive suicidal ideation if they lost access to GAC, for a total of 104 participants (21.3%).

**Table.** Sample Characteristics and Demographics

Characteristics	Participants, No. (%) (N = 489 participants)
Age, mean (SD), y	25.6 (6.3)
Sex assigned at birth	
Male	132 (27.0)
Female	337 (68.9)
Intersex	20 (4.1)
Gender identity	
Transgender man	127 (26)
Transgender woman	96 (19.6)
Nonbinary	187 (38.2)
Other (eg, demi-man, genderqueer)	79 (16.2)
Race^a^	
American Indian or Alaska Native	23 (3.3)
Asian	22 (4.5)
Black or African American	16 (4.7)
Native Hawaiian or Pacific Islander	2 (0.4)
White	459 (93.9)
Other^b^	27 (5.5)
Ethnicity	
Hispanic or Latinx	38 (7.8)
Not Hispanic or Latinx	451 (92.2)
Education^c^	
Less than high school	18 (3.7)
High school or General Educational Development	69 (14.1)
Some college, no degree	184 (37.6)
Associate degree	44 (9.0)
Bachelor’s degree	134 (27.4)
Master’s degree	33 (6.7)
Doctoral degree (eg, PhD, DNP, or EdD)	5 (1.0)
Professional degree (eg, JD or MD)	2 (0.4)
Region of the US	
Midwest	149 (30.5)
Northeast	95 (19.4)
Southeast	91 (19.2)
Northwest	76 (15.5)
Southwest	73 (14.9)
Missing	2 (0.4)

^a^ Participants could select more than 1 race, and the counts may not add up to 100%.

^b^ Other race refers to participant write-in responses of Cherokee (1), Hispanic (18), Inuit (1), and Middle Eastern (8).

^c^ Due to rounding the counts may not add up to 100%.

Box. **Examples of Participants’ Responses^a,b^**

**Item 1 Response Examples**

“I’m concerned about being unable to continue my hormone therapy through the VA, and not have it be covered.”
“I’m worried about a lot of access to all gender affirming care will be taken away under Trump. HRT therapy is giving me my life back.”
“Get medications internationally/from black market sources, or producing my own. None of which I’d like to do.”

**Item 2 Response Examples: DIY Hormones**

“DIY hormones are likely but in my eyes that is also a form of receiving care. Beyond which, I suppose the only other ‘cure’ to the problem would be death.”
“I’d try ordering e[strogen] online.”
“I’ll go underground for hormones.”
“Black/grey market hormones.”

**Item 2 Response Examples: Suicidal Ideation**

“I’ll kill myself. Or try to leave the country, but killing myself would be easier sooooo…” (Active suicidal ideation)
“If my access to top surgery is banned, I will kill myself. It is crucial to my mental health.” (Active suicidal ideation)
“I can’t go back in the closet. I will resume self-harming and hopefully die if I am forced back in.” (Passive suicidal ideation)
“Genuinely, I don’t think I will survive [being closeted] again. It is so mentally painful to imagine.” (Passive suicidal ideation)

^a^
Abbreviations: DIY, do-it-yourself; HRT, hormone replacement therapy; VA, Veterans Affairs.

^b^
Reponses are reported as the participant typed them.

## Discussion

In this survey study, we found that many gender minority individuals fear losing access to GAC in the next 4 years. Of our sample, 1 in 3 participants intended to access unregulated hormones, while 1 in 5 anticipated active or passive suicidal ideation if access to GAC is restricted. Both findings pose serious risks. Unregulated and unmonitored hormone use can cause health issues, while forced detransition can worsen mental health. Clinicians must anticipate and mitigate these harms by maintaining judgment-free, welcoming clinical engagement to offer support. GAC contributes to life satisfaction,^1,2^ which is associated with better mental health outcomes.^2,3,4^ Therefore, clinicians must advocate against restrictions on GAC to promote positive mental health outcomes. This study has limitations, including a cross-sectional design, excluding non–English-speaking US individuals, and a nonrepresentative sample skewed toward younger, non-Hispanic, White participants.

## Notes

Supplement 1. **eAppendix.** Supplemental Methods

Supplement 2.
Data Sharing Statement
