# Supplementary material for: Access to Gender-Affirming Care and Alternatives to That Care Among Transgender Adults
Source: JAMA Netw Open. 2025 Jul 16;8(7):e2520808. doi: 10.1001/jamanetworkopen.2025.20808 (PMC12268480; doi:10.1001/jamanetworkopen.2025.20808)
Supplement: Supplement 1. — eAppendix. Supplemental Methods [file jamanetwopen-e2520808-s001.pdf]

Supplemental Online Content

Graziano TA. Access to gender-affirming care and alternatives to that care among transgender adults. *JAMA Netw Open.* 2025;8(7):e2520808. doi:10.1001/jamanetworkopen.2025.20808

eAppendix. Supplemental Methods

This supplemental material has been provided by the authors to give readers additional information about their work.

© 2025 Graziano TA. *JAMA Network Open*.

eAppendix. Supplemental Methods

Participants

This study was approved by the University of Vermont IRB (Study 00003434). Participants had to be 18 years or old, fluent in English, identify as a transgender (i.e., transgender men and women, nonbinary individuals, or other non-cisgender identity) or intersex (self-identified), reside in the U.S., and plan to reside in the U.S. for at least 1 calendar year. Participants were excluded if they were cisgender. Paid advertisements were distributed across social media platforms (i.e., Tumblr, Reddit [r/Nonbinary, r/LGBTQStudies, r/TransHelpingTrans, r/TransMasc] Facebook, Bluesky, and Instagram). Responses were anonymous.

Data Collection

Informed consent was not required, and a waiver was granted by the UVM IRB. Instead, participants were first presented with an information sheet containing information about the study, including risks, benefits, and compensation information. It then asks if potential respondents agree to participate in the survey. If they indicated "yes," then the survey would begin. If they indicated "no" then they would be presented with a message thanking them for their interest and instructing them to close the window. Participants were asked to respond to the following questions:

Demographics. Demographic questions asked participants about their gender identity, sex assigned at birth, sexual orientation, race, ethnicity, age, education, income, and U.S. state or territory they reside in.

Open Response Item 1: "Do you think you will lose access to gender affirming care in the next four years?"

*Open Response Item 2**:*** "If you find yourself unable to receive gender-affirming care, what alternative options for care are you aware of or planning to take?"

Participants also answered other questions, but they are irrelevant to this report. Upon completion, participants were presented with contact information for suicide prevention resources and allowed to enter their email in a separate questionnaire to participate in a raffle for one of twenty $10 Amazon gift cards. Surveys took less than 15 minutes to complete on average.

Data Analysis

Data were downloaded and stored on an SPSS file, and demographics were analyzed using SPSS 29.0.2. Open response item 1 submissions were read in total and categorized as either believing they would lose access to GAC or not. For open response item 2, 0 = did not mention do-it-yourself hormone therapy, 1 = mentioned they would attempt do-it-yourself hormone therapy (i.e., using grey- and black-market hormones, sourcing hormones from out of state or country, and in some cases, biochemically produce their own hormone supply). This coding allowed us to count how many individuals were considering using unsecure hormone supplies. For Item 2, 0 = no mention of death, 1 = participant reported active suicidal ideation (e.g., "I would kill myself," or "suicide") if their access to care was restricted, and 2 = reported passive suicidal ideation (e.g., "I would not continue living," or "I would die"). This coding allowed us to count how many participants anticipate actively or passively having suicidal ideations should they lose access to GAC.

© 2025 Graziano TA. *JAMA Network Open*.
