# Supplementary material for: Access to Gender-Affirming Care and Alternatives to That Care Among Transgender Adults
Source: JAMA Netw Open. 2025 Jul 16;8(7):e2520808. doi: 10.1001/jamanetworkopen.2025.20808 (PMC12268480; doi:10.1001/jamanetworkopen.2025.20808)
Supplement: Supplement 2. — Data Sharing Statement [file jamanetwopen-e2520808-s002.pdf]

Data Sharing Statement

Graziano. Access to Gender-Affirming Care and Alternatives to That Care Among Transgender Adults. *JAMA Netw Open*. Published July 16, 2025. doi:10.1001/jamanetworkopen.2025.20808

Data
Data available: Yes
Data types: Deidentified participant data
How to access data: teresa.graziano@med.uvm.edu
When available: beginning date: 01-31-2026

Supporting Documents
Document types: None

Additional Information
Who can access the data: Researchers whose proposed use of the data has been approved
Types of analyses: For any purpose
Mechanisms of data availability: After approval of a proposal
